# Selective Th2 Upregulation by *Crocus sativus*: A Neutraceutical Spice

**DOI:** 10.1155/2011/639862

**Published:** 2010-09-13

**Authors:** Sarang Bani, Anjali Pandey, Vijai K. Agnihotri, Vijaylata Pathania, Bikram Singh

**Affiliations:** ^1^Cell Biology (Flowcytometry), Indian Institute of Integrative Medicine, Canal Road, Jammu Tawi 180001, India; ^2^Natural Plant Products Division, Institute of Himalayan Bioresource Technology (CSIR), Palampur 176061, India

## Abstract

The immunomodulatory activity of an Indian neutraceutical spice, saffron (*Crocus sativus*) was studied on Th_1_ and Th_2_ limbs of the immune system. Oral administration of alcoholic extract of *Crocus sativus* (ACS) at graded dose levels from 1.56–50 mg/kg p.o. potentiated the Th_2_ response of humoral immunity causing the significant increases in agglutinating antibody titre in mice at a dose of 6.25 mg/kg and an elevation of CD19^+^ B cells and IL-4 cytokine, a signature cytokine of Th_2_ pathway. Appreciable elevation in levels of IgG-1 and IgM antibodies of the primary and secondary immune response was observed. However, ACS showed no appreciable expression of the Th_1_ cytokines IL-2 (growth factor for CD4^+^ T cells) and IFN-*γ* (signature cytokine of Th_1_ response). A significant modulation of immune reactivity was observed in all the animal models used. This paper represents the selective upregulation of the Th_2_ response of the test material and suggests its use for subsequent selective Th_2_ immunomodulation.

## 1. Introduction

**Figure 1:**
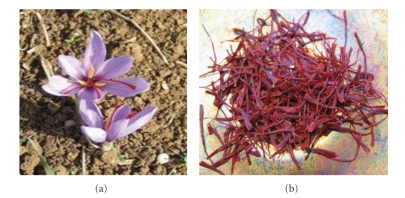
Crocus flowers and their reddish-orange stamens which are plucked, then dried.

Complementary and alternative medicines (CAM), together with traditional medicines, have attracted much attention [1]. The lesson to be learned and eventually put into practice concerns a wider approach to a search for useful drugs and applicable CAM products from diverse terrestrial sources [2–5]. There is even room to consider a diverse array of complex derivatives from certain natural products [6–9]. One theory of immune regulation involves homeostasis between T-helper 1 (Th_1_) and T-helper 2 (Th_2_) activity. Several nutrients and hormones measurably influence Th1/Th2 balance, including plant sterols/sterolins, oligodeoxynucleotides, probiotics, estrogen, and the minerals like zinc. In the present study we report the selective upregulation of the Th-2 response by, an Indian neutraceutical spice, saffron—*Crocus sativus* (Figure 1).

**Figure 2:**
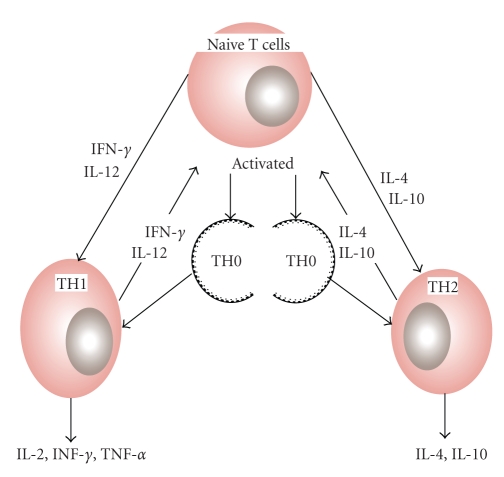
Cytokine-mediated differentiation of Th1/Th2 cells. Undifferentiated naive T cells secrete specific cytokines after antigenic stimulus that polarizes, first into null T-helper cells (Th0), then into Th1or Th2 cells.

Th_1_ cells follow the type-1 pathway (“cellular immunity”) to eliminate viruses and other intracellular pathogens, fight cancerous cells, and stimulate delayed-type hypersensitivity (DTH) reactions. Th_2_ cells drive the type-2 pathway (“humoral immunity”) and are responsible for the upregulation of the antibody production to fight extracellular pathogens; it is the type 2 dominance which is responsible for the tolerance of xenografts and of the fetus during pregnancy. T helper (Th) lymphocyte balance (Th1/Th2) is crucial in orchestrating the appropriate cytokine responses and hence remains as one of targets for immunomodulation and immune-based therapies. Th_1_-type cytokines (IFN-*γ*, IL-2) promote cell-mediated immunity and Th_2_-type cytokines (IL-4, 10) are responsible for humoral immunity leading to increased IgE. Over activation of either pattern can cause disease, and either pathway can downregulate the other. Hence, the optimal immunotherapy should restore or maintain a well balanced Th_1_ and Th_2_ response, suited to the immune challenge [10] (Figure 2). Saffron is a native of south Europe and is cultivated in Spain, France, Italy, Greece, Turkey, Persia, India, and China. In India it is one of the natural and delicious cash crop of Kashmir (Jammu and Kashmir state). It has been growing here since centuries and is cultivated on a commercial scale in the vast field in the Karewa lands of Pampore (5,300 ft above sea level). Botanically, saffron (*Crocus sativus*) belongs to a family of Iridacaee, is a small perennial herb with globular corms which attain a size of about 4 to 5 cm in diameter at maturity. Propagation of the saffron plant is through corms and is harvested from the dried, dark red stigmas of Crocus flowers. Since its tiny filaments must be painstakingly harvested by hand, it is no surprise that it ranks as the world's most expensive spice. Saffron has been widely used throughout the world due to its innumerable effect on the human body as a spice and as a folk medicine, to treat the nervous system (insomnia, paralysis), the respiratory system (asthma, colds), the cardiovascular system (heart disease), the digestive system (stomach disorders, flatulence, colic, and ailments such as scarlet fever, smallpox, gout, and eye disease. It has been also used as an aphrodisiac, antispasmodic, and expectorant [11]. Modern pharmacological studies have demonstrated *Crocus sativus* or its active constituents to have anti-inflammatory [12], antioxidant [13, 14], hypolipidemic [15], insulin-resistance reducing [16], and hypoglycemic [17] effects. Modulation of immune responses is, possibly, one most important factor responsible for these activities. Immune homeostasis is dependent on Th1/Th2 equilibrium. Shift towards Th1 or Th2 initiates abnormal condition related to many different disorders. As literature suggests saffron or its active constituents to have anti-inflammatory, antioxidant, hypolipidaemic properties, and so forth, these disorders are all related to proinflammatory Th1 pathway activation and their inhibition suggests Th2 upregulation induction by saffron. Th2 upregulation inhibits the hyperactivation of Th1 pathway thus balancing the Th1/Th2 paradigm.

## 2. Materials and Methods

### 2.1. Plant Material

The orange-red colored threelobed fresh stigmas of *C. sativus* Linn. were collected directly from farmers field of Srinagar, Jammu, and Kashmir, India and authenticated by Dr B. K. Kapahi (Indian Institute of Integrative Medicine (CSIR), Jammu and Kashmir, India). The collected stigmas (saffron) were dehydrated in hot air dryer at 40 ± 5°C (one batch) and was stored at 4°C under nitrogen for further experiments.

### 2.2. Preparation of Aqueous Extract from Dried Stigmas

The dried stigmas (10 g.) were extracted with deionized water under ultrasonic extractor (Sonics, Model VC 750) using amplitude 65% for 5 minutes without using pulse, for three times with maceration for complete extraction. The extract solution was filtered and freeze-dried (Telstar, Lyobeta 35) when it afforded a bright red coloured shining powder (6.5 g) which was stored at −4°C under nitrogen atmosphere for further experiments.

### 2.3. Column Chromatography for Isolation of Crocin-1 and Crocin-2

Marker compounds of the obtained extract were isolated by silica gel column chromatography (extract: silica gel ratio 1 : 20, elution solvent system (isocratic) : ethylacetate : methanol : water 100 : 16.5 : 13.5). The fractions were pooled according to TLC on solvent system EtOAc : MeOH : H_2_O (100 : 16.5 : 13.5) to afford crocin-1 (all-trans-crocetin di(*β*-D-gentiobioside ester)) and crocin-2 (all-trans-crocetin-*β*-D-gentiobiosyl-*β*-D-glucosyl ester) for the standardization of the extract [18].

### 2.4. HPLC Fingerprint for Analyzing the Saffron Extract

**Figure 3:**
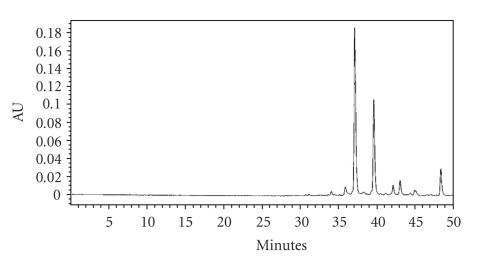
HPLC profile of water extract of ACS at 440 nm.

HPLC fingerprint was used to analyze the saffron extract. Balance (GH-252, MAX 210 g, d = 0.1 mg, produced by A & D Company Limited) and PDA-detector (water's 996 photodiode array detector) were used. The waters HPLC system equipped with pump (waters 600 quaternary gradient pump) with waters 717 plus automated sample injector facility, and empower 2 software. The column was stainless-steel column Lichrosphere 100-RP-18e (250 mm × 4 mm × 5 *μ*m; Merck Co., Ltd). The chromatographic conditions include mobile phase, the linear gradient from 10% to 100% methanol in water containing 1% acetic acid in 50 min. 10 minutes equilibration. The solvent flow rate was 1.0 ml/min and the sample injection volume 10 *μ*l (concn. 2 mg saffron/ml., Millipore Millex Nylon (0.45 *μ*m) filter was used for filtration) with PDA detector at wavelength of 440 nm (for Crocin). Crocin-1 eluted at 37.446 min while crocin-2 at 39.903 min (Figure 3).

### 2.5. Animals

Male Balb/c mice 10–12 week old and weighing 20–24 grams obtained from animal house of Indian Institute of Integrative Medicine, Jammu were taken up in groups of six and employed for the study. These animals were maintained at a room temperature of 20 ± 2°C with 12 h light/dark cycle with free access to pellet food and water. According to ethical regulations on animal research all animals used in experimental work received humane care. Animals were housed and maintained following standard guidance as found in Government of India guidelines [19]. The study protocol was approved by Institutional Animal Ethics Committee. Ten experimental groups comprising of six animals each were employed for the study including a naïve control group, sensitized control group, groups receiving standards, and drug-treated groups (receiving test drugs at graded concentration).

### 2.6. Drugs

Test drug was prepared in distilled water and was administered orally daily once a day for the duration of experiment at 1.56, 3.12, 6.25, 12.5, 25, and 50 mg/kg p.o. dose. The broad range of oral dosage was taken up to get dose reponse and to determine the dose with most significant effect. Preliminary study with the extract (data not shown) showed the significant effect to be at the doses of 6.25 and 12.5 mg/kg p.o. Keeping this in mind we carried out studies taking two lower and two higher doses along with the effective doses. Levamisole at 2.5 mg/kg per oral dose was used as positive control and Cyclosporin-A was used as immunosuppressive agent at 5 mg/kg per oral dose. The standards were also freshly prepared and administered orally during the experimental duration.

### 2.7. Antigenic Stimulus

Fresh sheep red blood cells (SRBC) were collected aseptically from the jugular vein of sheep and stored in cold sterile Alsever's solution These were washed three times with pyrogen free sterile saline (0.9% NaCl w/v) and adjusted to the concentration of 5 × 10^9^ cells/ml for immunization and challenge at the required time schedule.

### 2.8. Effect on General Behavior and Maximum Dose Tolerance in Mice

The acute oral toxicity studies were carried out after approval from the Institutional Animal Ethics Committee following OECD (Organization for Economic Cooperation and Development) Guidelines 423 [20]. The test material was found to be safe up to 2000 mg/kg with no adverse effects on general behavior in treated BALB/c mice.

### 2.9. Humoral Antibody Response to SRBC

Groups of 6 mice each were immunized by injecting 0.2 ml of 5 × 10^9^ SRBC/ml intraperitoneally (i.p.) on day 0 and challenged 5 days later on day 6 by injecting an equal volume of SRBC intraperetoneally. Blood samples were collected on day 7 for antibody titre. Haemagglutination antibody titres were determined following the microtitration technique described by Nelson and Mildenhall [21]. The value of the highest serum dilution causing visible haemagglutination was taken as a titre. BSA-saline alone served as a control.

### 2.10. Delayed Type Hypersensitivity Response (DTH)

The method of Doherty [22] was followed. 200 *μ*l of 5 × 10^9^ SRBC/ml were injected intraperitoneally to immunize the mice on Day 0. Six days later the thickness of the left hind food was measured with a spheromicrometer (0.01 mm pitch) and was considered as a control. The mice were then challenged by injecting the same amount of SRBC intradermally into the left hind footpad. The foot thickness was measured again after 24 h.

### 2.11. Plaque Forming Cell (PFC) Assay

Mice were immunized by an injection of 0.2 ml of 10% (v/v) suspension of SRBC in normal saline. Five days later the animals were sacrificed and their spleen was removed aseptically. A single cell suspension of an individual spleen was prepared in RPMI 1640 medium and viability was checked by trypan blue dye exclusion test. Cell concentration was adjusted to 2 × 10^7^ cells/ml in RPMI 1640. To 0.1 ml of this suspension, 0.3 ml of 5% SRBC suspension, 0.l ml of guinea pig serum, and 0.5 ml of RPMI 1640 medium were added. A 150 *μ*l quantity of this mixture was added to each Cunningham's chamber. The chambers were sealed with paraffin wax to prevent dehydration. These chambers were incubated for 60 to 90 min at 37°C in incubator. Plaques in SRBC lawn were enumerated using an inverted microscope. Each plaque represented a product of an individual antibody forming cell [23].

### 2.12. Lymphocyte Immunophenotyping

**Figure 4:**
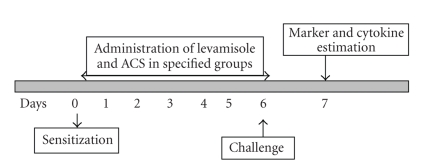
Schematic representation of experimental procedure.

Immunizatin of Balb/c mice was carried out by injecting 200 *μ*l of 5 × 10^9^ SRBC/ml i.p. Drug administration was carried out for 5 days. Same amount of SRBC was then injected into the mice for the challenge and blood was collected after 48 h of challenge in heparinised tubes from retroorbital plexus (Figure 4). Lymphocyte subsets were measured by immunofluorescent antibody staining of whole blood and subsequently analyzed using two color flow cytometry. Murine monoclonal antibodies conjugated to a fluorochrome and directed against receptors CD3, CD4 and CD8 were used for the study. FITC (Fluoroisothiocyanate) labelled antimouse CD4 monoclonal antibody that reacts with the CD4 surface antigen expressed on MHC class II restricted T cells that includes most helper cells and FITC (Fluoroisothiocyanate) labelled CD8 monoclonal antibody that reacts with CD8 differentiation antigen present on MHC class-I restricted T cells were used to determine the percentage of CD4^+^ and CD8^+^ T cells in control and treated groups of animals. For the B cell counts (TH2 response) CD19^+^ antimouse monoclonal antibody was used which was labeled with the Phycoerythrin flourochrome. These fluorochrome-labeled monoclonal antibodies were added directly to 100 *μ*l of whole blood, which was then lysed using whole blood lysing reagent (BD Biosciences). Following the final centrifugation, samples were resuspended in phosphate-buffered saline (pH, 7.4) and analyzed directly on the flow cytometer (LSR, BD Biosciences) using Cell Quest Pro Software (BD Biosciences) [24, 25].

### 2.13. Intracellular Cytokine Estimation (Th1 and Th2 Cytokines)

The blood was collected in heparinised tubes from retroorbital plexus as mentioned above. Fluoroisothiocyanate, FITC-labeled antimouse IL-2 monoclonal antibody and Phycoerythrin (PE)-labeled IL-2 monoclonal antibodies were used in one set and Fluoroisothiocyanate, FITC-labeled antimouse labeled IFN-*γ* monoclonal antibody was used in the other set of experimentation, however, for the Th2 response PE labeled IL-4 was used [24, 25].

### 2.14. Evaluation of Primary Ig-G and Ig-M Antibody

The analysis of subsets namely Ig-G and Ig-M was performed on peripheral blood. Briefly, mice were bled at required time schedules and 50 *μ*L of blood was added to falcon tubes (BD Biosciences) containing different antimouse immunolabeled monoclonal antibodies for Ig-G1 and Ig-M. After mixing and incubating at room temperature for 30 minutes in the dark, FACS lysing solution was added. The samples were incubated for 10 minutes at room temperature, followed by centrifugation. The cells were washed and enumeration of lymphocytes subsets was done using flow cytometer. 10,000 events were collected to analyze Ig-G and Ig-M expression [24, 25].

### 2.15. Statistical Analysis

The results were expressed as Mean ± SEM of three independent experiments. Results are presented as fold increase/decrease values compared with the untreated control. Statistical significance was determined by Student's *t*-test, comparing the experimental group to the control group.

## 3. Results

### 3.1. Effect on General Behaviour and Maximum Dose Tolerance in Mice

Mice treated with ACS at a maximum oral dose of 2000 mg/kg did not show any difference in gross general behaviour compared with the control group of animals that were administered only the vehicle. No mortality was observed over an observation period of 7 days.

### 3.2. Humoral Antibody Response to SRBC

As indicated in Table 1 ACS modulated the humoral immune response and showed a dose-dependent increase in antibody titres. Significant increase in antibody titres was observed at 6.25 mg/kg (*P* < .001), under the same conditions, significantly higher antibody titres were observed with Levamisole at dose of 2.5 mg/kg (*P* < .001). The immunomodulatory activity of ACS at 6.25 mg/kg observed was 29.93%.

### 3.3. Delayed Type Hypersensitivity Response (DTH)

The DTH response showed by ACS is reflected in the CMI Response in Table 1. ACS when administered orally showed a dose-dependent increase where maximum effect was observed at 6.25 mg/kg p.o. (*P* < .01) where the immunomodulatory effect on the potentiating side was found to be 14.70%. Levamisole that was used as a positive standard at dose of 2.5 mg/kg resulted in higher immunomodulating activity (*P* < .001) with the percentage increase of 29.14% in normal Balb/c mice, however, Cyclosporine A markedly suppressed paw oedema where a decrease of 38.23% was observed.

### 3.4. PFC Assay

**Figure 5:**
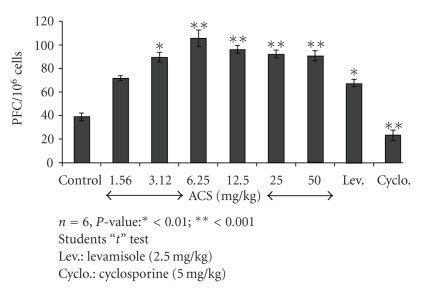
Effect of ACS on humoral immunity as assessed by PFC assay.

The effect of ACS on the number of PFC in the spleen is shown in Figure 5. The maximum increase in the PFC assay in ACS treated groups at 6.25 mg/kg was observed on day 5 after the immunization in comparison with control animals. Levamisole (2.5 mg/kg) used as a positive control produced significant stimulation in the PFC assay.

### 3.5. Lymphocyte Immunophenotyping

The animals treated with ACS at 6.25 mg/kg p.o. dose-dependent effect of 33.26% for CD4^+^ and 17.40% for CD8^+^ T cells and 32.90% of CD4^+^ and 17.16% of CD8^+^ T cells at per oral dose of 12.5 mg/kg. In the sensitized control group the percentage of T lymphocyte subsets observed was 26.25% and 19.66% for CD4^+^ and CD8^+^ T cells. Cyclosporin-A a standard immunosuppressive T cell inhibitor at 5 mg/kg oral dose inhibited both CD4^+^ and CD8^+^ T cells showed 18.61% of CD4^+^ and 10.29% of CD8^+^ T cells. Levamisole potentiated both CD4^+^ and CD8^+^ T cell counts, the increase observed was 37.30% and 21.50% for CD4^+^ and CD8^+^ T lymphocyte subsets. 

**Figure 6:**
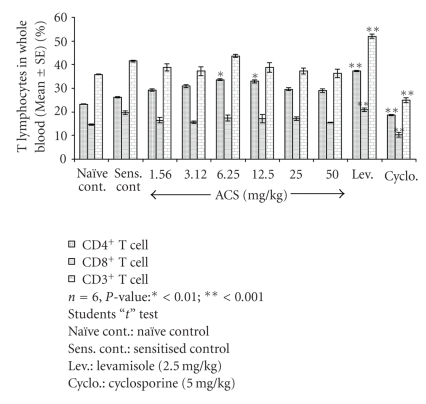
Effect of ACS on T lymphocytes (CD4^+^, CD8^+^, CD3^+^) in normal Balb/c mice.

As indicated in Figure 6, ACS showed a dose-dependent increase in CD3^+^ T cell subsets, which represent the total “T” cell population. ACS at 6.25 mg/kg dose showed 43.70% of CD3^+^ T cell counts and 38.79% at 12.50 mg/kg. Cyclosporin inhibited CD3^+^ T lymphocytes and Levamisole significantly increased the total lymphocyte counts where 24.90% was observed for Cyclosporin and 52% was observed for Levamisole.

**Figure 7:**
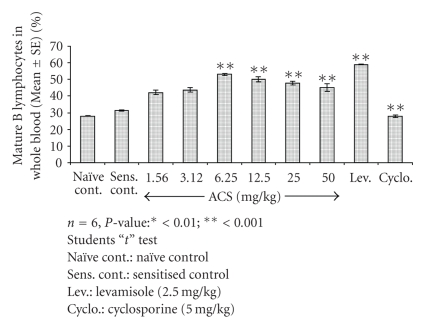
Effect of ACS on B lymphocytes (CD19^+^) in normal Balb/c mice.

The effect of test material on CD19^+^B cell counts; representing the B cell population of the Th2 response was studied in normal Balb/c mice (Figure 7). Test material administration at 6.25 mg/kg resulted in a significant increase in CD19^+^B cells (*P* < .001) where the percentage observed was 53%, however, for the sensitized control group 31.40% of B lymphocytes was observed. Cyclosporin-A administration resulted reduction in B cell counts (*P* < .001) as compared to control indicative of immune suppression and Levamisole significantly increased the B cell types of the Th2 response.

### 3.6. Effect of ACS on Th1 and Th2 Cytokines

To determine immunomodulating effect on Th1-Th2 balance, selected cytokine levels produced by Th1 and Th2 subsets were measured *in vivo* using flow cytometry.

### 3.7. Th1 Cytokines (IFN-Gamma and IL-2)

**Figure 8:**
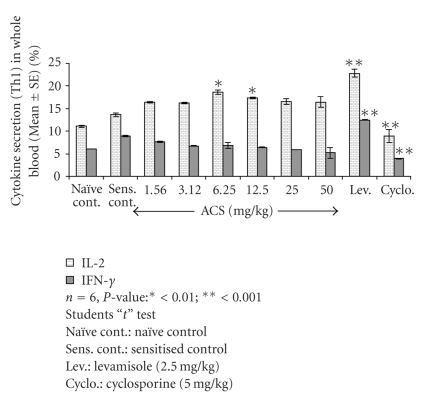
Effect of ACS on Th1 cytokine (IL-2, IFN-gamma) expression in whole blood of normal Balb/c mice.

Oral administration of ACS at specific doses showed a significant dose dependent upregulation of Th1 cytokine IL-2, growth factor for CD4^+^T cells at the oral dosage of 6.25 mg/kg as compared to sensitized control. (*P* < .001), percent IL-2 expression observed was 18.60% for ACS however in sensitized control group it was evaluated to be 13.63%., but for IFN-*γ* no significant increase was observed. Levamisole significantly upregulated IFN-*γ* production, which was observed to be 12.46%. Interestingly, IFN-*γ* was downregulated at 25 and 50 mg/kg p.o dose (Figure 8) in which the percentages dropped to 5.90% and 5.20%.

### 3.8. Th2 Cytokine (IL-4) Expression

**Figure 9:**
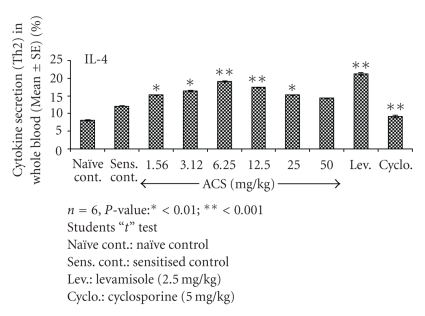
Effect of ACS on Th2 cytokine (IL- 4) expression in whole blood of normal Balb/c mice.

A significant effect of ACS was observed on IL-4 levels as compared with sensitized control, significant higher IL-4 levels were observed for Levamisole (*P* < .001). At 6.25 mg/kg and 12.50 mg/kg the IL-4 expression was observed to be 19.10% and 17.40%, when compared to sensitised control in which 12.08% of IL-4 was observed. The increase in expression levels of IL-4 by ACS can be compared to Levamisole in which 21.30% of IL-4 secretion was observed (Figure 9). This suggests ACS to possess selective modulating property of Th1-Th2 balance of the immune system.

### 3.9. Evaluation of Primary Ig-G and Ig-M Antibody Expression

**Figure 10:**
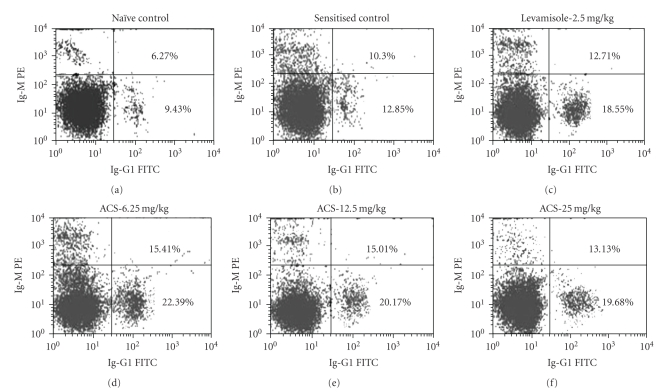
Flow cytometric representation showing effect of ACS on Ig-G1 and Ig-M. Cells were labeled with Ig-G1 (FITC-conjugated monoclonal antibody) and Ig-M (PE-conjugated monoclonal antibody) and analysed in flowcytometer using cell-quest software.

Elevation in levels of IgG-1 and IgM antibodies of the primary and secondary immune response was observed in ACS treated groups (Figure 10).

## 4. Discussion

Many herbal preparations alter immune function and display an array of immunomodulatory effects. In various *in vitro *and *in vivo *studies, herbal medicines have been reported to modulate cytokine secretion, histamine release, immunoglobulin secretion, class switching, cellular coreceptor expression, lymphocyte expression, phagocytosis, and so on [26, 27]. Modulation of the Th1/Th2 balance by administration of recombinant cytokines or cytokine antagonists alters the outcome of the diseases [28]. However, their clinical efficacy has been limited and has associated complications [29, 30]. The trends indicate that there is an utmost need for orally active nonpeptide compounds that can modulate Th1/Th2 balance [31]. In the present study, we report that “ACS” dose-dependently potentiates the DTH reaction induced by SRBC. DTH is a part of the process of cell-mediated immune response and requires the specific recognition of a given antigen by activated T lymphocytes, which subsequently proliferate and release cytokines. These in turn increase vascular permeability, induce vasodilatation, macrophage, and activation, promoting increased phagocytic activity [32]. Increase in DTH reaction in mice in response to antigen revealed the stimulatory effect of ACS on T lymphocytes and accessory cell types required for the expression of reaction [33]. Helper T (Th) cells are a crucial component of the adaptive immune system and are of fundamental importance in orchestrating the appropriate response to pathogenic challenge. They fall into two broad categories definned by the cytokines each produces. Th1 cells produce interferon-gamma (IFN-*γ*) and are required for selective immunity to intracellular bacteria, viruses, and protozoa whereas Th2 produce IL-4 and are required for optimal antibody production to T-dependent antigens. Functionally, Th1 lymphocytes are associated with inflammation and cell-mediated responses, whereas Th2 cells provide help to B cells and so are linked with antibody production. Infections that can be cleared by type 1 responses include mycobacterial infections such as tuberculosis and, *Leishmania major*, and viruses such as influenza; Th1 cells are also associated with autoimmune diseases and graft rejection. Th2 responses, on the other hand, give protection against helminths, certain viral infections (e.g., measles) and are strongly associated with allergy. The present study indicated that ACS showed a dose-dependent increase in the T cells-mediated DTH reaction in mice (Table 1). This showed its potentiating effect “T” lymphocytes [34]. Evidence that lends support to the hypothesis of T-lymphocytes potentiation is the significant potentiating effect of ACS on CD4^+^ and CD8^+^ T cells (Figure 5). T Cells expressing CD4 are increased when there is a general expansion due to active immunological activity of the T cell lymphocyte subsets. Cytokines play important roles at different stages of the immune response, and indeed are usually multifunctional. For example, with sufficient antigenic stimulus, IFN-*γ* and IL-2 are important for differentiation and proliferation of Th1 cells whereas later their presence serves to enhance apoptosis of repeatedly stimulated effector cells. The Th1 and Th2 proliferation rates are simple functions of the concentrations of the growth factors IL-2 and IL-4, as well as of IFN-*γ*. ACS induced a dose related upregulation of IL-2 (interleukin-2) production by CD4^+^ T cells (Figure 7). IL-2 is secreted by stimulated helper T cells and cytotoxic T cells. It promotes proliferationand differentiation of additional CD4^+^ cells, B cells, and activates macrophages. But when compared to Levamisole (positive standard) IL-2 and IFN-*γ* were not significantly upregulated. The humoral immunity involves interaction of B cells with the antigen and their subsequent proliferation and differentiation into antibody-secreting plasma cells. Antibody functions as the effector of the humoral response by binding to antigen and neutralizing it or facilitating its elimination by cross-linking to form clusters that are more readily ingested by phagocytic cells. To evaluate the effect of ACS on humoral response, its influence was tested on sheep erythrocyte-specific haemagglutination antibody titre in mice. Cyclosporin at a dose of 5 mg/kg, p.o., showed significant inhibition in antibody titre response, while ACS was found to significantly enhance the production of circulating antibody titre. Since “ACS” augmented the circulating antibody titre, it was thought worthwhile to evaluate its effect effect on CD19 ^+^B cells, IL-4 and to study the IgG1 and IgM response of the primary and the secondary reponse of the immune system by Flowcytometry. ACS showed significant enhancing effect of CD19^+^ B-lymphocytes where approximately a two-fold increase was observed, which was comparable to Levamisole. ACS highly upregulated the expression of IL-4 cytokine, which is an autocrine growth and differentiation factor for Th2 cells, the increase in IL-4 level was highly comparable to the IL-4 secretions of Levamisole. Furthermore, the results presented herein show that ACS treatment increased two to three fold the production of anti-SRBC IgG and IgM lymphocytes from immunized mice when measured by flowcytometry (Figure 9).

The result suggests that the oral administration of ACS drives a shift in Th1 : Th2 balance toward Th2-dominant immunity by increased IL-4 cytokine expression levels rather than Th1 reponse. Levamisole up regulated Th1 as well as Th2 cytokines (Figures 7 and 8).

## 5. Conclusion

In conclusion, this study suggests that even complex botanical mixtures can exhibit selectivity in immune therapy and will be useful to underline importance of systems approaches in the ethnopharmacology based drug discovery [35, 36]. This findings outlined above establish Th2 upregulating activity of ACS and suggests its use in conditions where Th1/Th2 modulation is required and thus is suggestive of its possible therapeutic usefulness. Such plant based modulators may have applications in the treatment of immunodeficiency diseases, allergic manifestations, and for combinational therapy with antibiotics and as vaccine adjuvants. Its apparent safety over long-term administration is encouraging enough to warrant further studies to explore its possible therapeutic role in modern clinical practice.

## Figures and Tables

**Table 1:** Immunomodulatory activity of ACS on Humoral (Antibody titre) and cell-mediated immune response (CMI) in normal Balb/c mice.

Test material	Dose mg/kg p.o.	Humoral response antibody titre mean ± S.E.	CMI response foot thickness (mm) 24 h mean ± S.E.
Control	—	6.18 ± 0.36	0.68 ± 0.06
ACS	1.56	7.03 ± 0.11 (13.91↑)	0.74 ± 0.28 (8.82↑)
ACS	3.12	7.10 ± 0.28 (14.88↑)	0.75 ± 0.38 (10.29↑)
ACS	6.25	8.03 ± 0.43** (29.93↑)	0.78 ± 0.30* (14.70↑)
ACS	12.50	8.01 ± 0.56** (29.61↑)	0.77 ± 0.13 (13.23↑)
ACS	25.00	7.63 ± 0.48** (23.46↑)	0.76 ± 0.43 (11.76↑)
ACS	50.00	7.40 ± 0.56* (19.74↑)	0.73 ± 0.48 (7.35↑)
Levamisole	2.5	8.39 ± 0.16** (35.76↑)	0.88 ± 0.18** (29.14↑)
Cyclosporine	5.00	3.92 ± 0.31** (36.56↓)	0.42 ± 0.13** (38.23↓)

Value in parentheses signify percentage activity in agglutinating antibody titre and CMI Response

↓ : Percent Decrease, ↑ : Percent Increase

*n* = 6,

All values are shown as Mean ± S.E.

*P*-value:* < .01;** < .001,

*P-*values were calculated by Student's *t*.
